# Advertising That Makes for Ill-Health

**Published:** 1917-01

**Authors:** 


### ADVERTISING THAT MAKES FOR ILL-HEALTH.

One phase of the problem of "patent medicine" advertising is frequently lost sight of. The modern advertiser, in any line, is not so much concerned with letting the public know that he can supply what it legitimately demands, as he is in creating an artificial demand which he hopes to supply. As an advertising concern in Chicago says, in a circular letter sent to prospective clients:

"It would be a liberal estimate to say that only twenty-five per cent. of the business transacted in this country each day is done as the result of a 'natural demand.' The other seventy-five per cent. is done as a result of salesmanship in one form or another—and it's on the seventy-five per cent. that we make our living and you make yours."

This, we believe, is no exaggeration. It means, in other words, that three-fourths of the things purchased are bought not because the public normally wants them, but because, through skillful advertising, the public has been hypnotized into believing it wants them. It may be that in many lines of business this is legitimate. At any rate, it is conceivable that some reasonably convincing arguments might be made in favor of it. But in the matter of selling drugs for the self-treatment of ailments, such artificially created demands are not legitimate. They are directly against public interest because they are a menace to the public health.

The real object of "patent medicine" advertising—as all "patent medicine" advertisers know, although few will admit it—is not the simple one of telling the public what goods there are for sale. It has a much more subtle motive. Its real intent is to convince those who read the advertisement that they are suffering from certain ailments which can be cured by the preparation advertised. One "patent medicine" maker who was urging druggists to stock his "cure" for appendicitis, and presumably thought that he could be frank about it, said that unless his product was put on the shelf the druggist would have nothing to sell "to the man who has appendicitis nor to the vast multitude who THINK they have or are going to have this dread disease." Elaborating, the manufacturer reminded the druggist further, that "fully seventy-five per cent. of all cough and kidney remedies are bought by people who THINK they have consumption or some serious kidney ailment . . . and not by people who actually have them." That's it exactly! And it is the business of the "patent medicine" advertisement to play on the fears of those who are temporarily indisposed and make them think that they have this, that or the other disease, which can surely be cured by Dr. Quack's Panacea.

What, then, is the remedy? That no medicine should be sold for the self-treatment of ailments? By no means. Were we living under ideal economic conditions, it might be as feasible as it may be theoretically desirable, that expert advice and opinion should be sought whenever anything went wrong with the human machine. Under present economic conditions, however, such a conception is Utopian. It is unthinkable that the average man is going to seek a physician's aid every time he becomes constipated. Instead, he is going to purchase a laxative medicine of some sort. It may be admitted, then, that there is today a legitimate place on the market for home remedies for the self-treatment of simple ailments. All that has been asked in the interest of public health and safety is that these remedies be sold under no misleading claims; that they contain no dangerous or habit-forming drugs, and that the names and amount of the active ingredients therein be declared on the label.

The public has a further right in the premises, although it has not awakened to this fact. It is justified in demanding that such remedies shall not be so advertised as to make for ill-health. "Patent medicine" advertising has for years been a stench in the nostrils of thinking men, lay and medical. It has been the black beast of the advertising world. Every effort made by decent advertisers in other lines of endeavor toward purifying the advertising field has had first to be directed against the "patent medicine" business. Yet, from the point of view of the public health, the advertising of home remedies, instead of being the most exaggerated, the most suggestive and the most fraudulent, should, on the contray, be even more conservative and respectable than the advertising of the ordinary products of commerce. It may or may not be a bad thing for the community, economically speaking, if the public is led, through skillful advertising, to buy more hats, more pianos or more automobiles than it can really afford or has any legitimate use for. But it requires no argument to prove that advertising which makes a well man think he is sick and a sick man think he is *very sick,* with the object in view of making these men drug themselves unnecessarily, is a crime against the public health.

